# Use of the Smart Lean Method to Conduct High-Quality Integrated Perioperative Management Prior to Hospitalization

**DOI:** 10.3390/ijerph182413391

**Published:** 2021-12-20

**Authors:** Hung-Wen Tsai, Su-Wen Huang, Yin-Lurn Hung, Yu-Shan Hsu, Chien-Chung Huang

**Affiliations:** 1 Department of Medical Administration, Taichung Veterans General Hospital, Taichung 40705, Taiwan; tsaihw@vghtc.gov.tw (H.-W.T.); hyluvn@vghtc.gov.tw (Y.-L.H.); hsuyushan@vghtc.gov.tw (Y.-S.H.); 2 Division of Cardiovascular Surgery, Taichung Veterans General Hospital, Taichung 40705, Taiwan; 3 Department of General Affairs, Taichung Veterans General Hospital, Taichung 40705, Taiwan; 4 Department of Information Management, Chaoyang University of Technology, Taichung 41349, Taiwan; 5 Department of Computer & Communications Center, Taichung Veterans General Hospital, Taichung 40705, Taiwan; ccwhung@vghtc.gov.tw

**Keywords:** Lean and Six Sigma methodologies, management waiting time, optimize the admission process

## Abstract

Background: competition in the healthcare market is becoming increasingly intense. Health technology continues to evolve, so hospitals and clinics need to strengthen hospital management techniques and also adopt a more patient-centered approach in order to provide high-quality healthcare services, including a more simplified process and shorter waiting times for examinations. The Lean and Six Sigma methodologies and smart technology were introduced and implemented into the integrated perioperative management (PERIO) processes for the purpose of decreasing pre-admission management waiting time, as well as increasing the completion rate and quality of pre-admission management for surgical patients in a 1576-bed medical center in central Taiwan. Methods: in order to improve hospital admission procedures for surgical patients by shortening process waiting times, simplifying admission processes, emphasizing a patient-centered approach, and providing the most efficient service process, the present study applied the DMAIC architecture of the Lean Six Sigma. This approach allowed the patients to save time on the hospital admission process. The current workflow used value flow mapping to identify wasted time caused by unnecessary walking and waiting during the hospital admission process. Therefore, we improved the process cycle for each patient by simultaneously selecting and controlling the process for the purpose of saving time. Results: the experimental results show that the percentage of Process Cycle Efficiency (PCE) increased from 35.42% to 42.47%, Value Added was reduced from 34 to 31 min, and Non-Value Added was reduced from 62 to 42 min. The satisfaction score of the 97 pre-implementation patients was 4.29 compared with 4.40 among the 328 post-implementation patients (*p* < 0.05). The LOS (Length of Stay) of 2660 pre-implementation patients was 2.49~3.31 days and for 304 after-implementation patients it was 1.16~1.57 days. Conclusions: by integrating different units and establishing standard perioperative management (PERIO) procedures, together with the support of the information systems, the time spent by patients on hospital admission procedures was shortened. These changes also improved the comprehensiveness of the preoperative preparations and the surgical safety of patients, thereby facilitating the provisions necessary for high-quality healthcare services. This in turn reduced the average length of hospital stays and increased the turnover of patients, benefiting the overall operations of the hospital.

## 1. Background

Competition in the healthcare market is becoming increasingly intense. Health technology continues to evolve, so hospitals and clinics must strengthen hospital management techniques and also adopt a more patient-centered approach in order to provide high-quality healthcare procedures, including shorter waiting times for examinations and a more simplified process. Thus, it is important to optimize the service processes which patients must go through.

Lean methods enhance Value Added (VA) efficiencies, improve on time wasting processes, create well-defined and realistic targets, and measure outcomes of specific improvements. Not only is this approach far more cost-effective than a complete system redesign, it also capitalizes on the inherent strengths of the existing VA infrastructure [1].

Throughout the surgical experience, patient safety is a critical aspect of a hospital’s performance. Appropriate completion of the preoperative assessment of the patient, known as a time out protocol, is often the main mechanism utilized in a surgical environment to ensure patient safety [2].

The present study referred to the Inpatient Satisfaction Survey Report of TCVGH during the years 2015 to 2017 (Table 1). This questionnaire was designed to gain a better understanding of patients’ satisfaction with waiting times, provision of healthcare information, quality of healthcare services, and the environmental facilities during their care. The items related to this study were Q6, “Processes for various examinations during hospital admission” and Q18, “Convenience of hospital admission procedures at the Admission Service Center”. The results of the six most recent reports show that the level of satisfaction for “Processes for various examinations during hospital admission” was lower than the average level of satisfaction for this dimension, and that there also remained room for improvement in the level of satisfaction for “Convenience of hospital admission procedures at the Admission Service Center”.

Based upon the above data, we determined that the main problem was that the patients were waiting too long to complete the hospital admission procedure. We used a cause and effect diagram to analyze and discuss the following problems encountered by patients during the various processes they went through for hospital admission:

(1). Examination units are spread out, so patients spend a lot of time walking between units.
(2). The poor walking routes in the hospital admission process cause patients to walk back and forth between the same units.
(3). The manpower at the Admission Service Center counter is unable to handle the number of patients during peak times, thus increasing patient waiting times.
(4). There exists a failure to make good use of information systems and equipment, as well as a failure to synchronize information in real time and make full use of equipment.

Using field research, the present study explored the processes and time spent on testing and examinations, as well as hospital admission procedures for patients prior to admission to Taichung Veterans General Hospital (TCVGH). This study used a questionnaire to collect patient satisfaction data and to also identify waiting times and areas of dissatisfaction (for example, complicated procedures, departments which are far apart, failure to make effective use of information systems and equipment, and failure to synchronize information in real-time and make full use of equipment). The present study aimed to improve procedures using sophisticated methods in order to shorten the processing times for hospital admission. At the same time, smart technology and information services were introduced into the pre-admission examination procedures to improve the completeness of pre-admission examinations, so that patients and family members could complete the hospital admission procedure in the most convenient and efficient way. The new process has been designed to improve a hospital’s service quality and patients’ satisfaction [3,4,5,6,7].

To understand the time spent on, and the detailed processes of, hospital admission procedures and examinations prior to admission, the present study used field research taken from the perspective of patients. This entailed accompanying patients during the hospital admission process and recording the time spent on each process for purposes of data analysis and obtaining statistics, as well as understanding patient satisfaction through the use of a questionnaire.

Our results show that the perioperative system decreased the time necessary from admission to surgery, and that it is useful in providing a high-quality medical service, although the system should be improved in a way so as not to increase any burden on medical staff. Perioperative management (PERIO) of surgery patients is critical for improving the overall outcomes of surgeries. The perioperative system has been shown to successfully reduce the time needed from admission to surgery. In addition, it may help prevent or reduce the incidence of surgery cancellation [8,9,10].

## 2. Methods

### 2.1. Lean Six Sigma

Lean Six Sigma is a quality improvement tool, as well as a management activity, that is used to improve corporate performance. The concept of Lean Six Sigma was introduced by Motorola in 1985. Because the company’s product defect rate was too high, which impacted its market share, the company developed an improvement plan as its basis for quality improvement and greater production.

This was subsequently introduced as a standard for quality improvement by the General Electric Company in 1995, incorporating the original process into defined steps to perfect the process and develop the DMAIC (Define, Measure, Analyze, Improve, Control) model. The DMAIC model is divided into five stages (Table 2).

In order to improve hospital admission procedures for surgical patients by shortening process-waiting admission times, simplifying the admission process, emphasizing a patient-centered approach, and providing the most efficient service process, the present study applied the DMAIC architecture of the Lean Six Sigma. At the same time, the present study used value-flow mapping to identify wasted time caused by both unnecessary walking between procedures, as well as the waiting time during the hospital admission process. We then calculated both the Process Cycle Efficiency (PCE) and Process Cycle Efficiency Destruction (PCED) for each process, selecting and controlling the process improvements to shorten the time spent by patients on the hospital admission process [11,12,13].

### 2.2. Perioperative Management (PERIO)

Inpatient surgical patients require integrated assessments. With an increasing age of patients and the complexity of their conditions, patients need to have blood tests, ECG tests, and chest X-rays taken after the physician has decided on the surgical procedure. Comprehensive preoperative assessments and health education are required so that anesthesiologists and surgeons can provide the best surgical results. Comprehensive preoperative assessments are performed to quickly find abnormal test values and then correct them. For special surgical groups, in order to avoid excessive bleeding during surgery, a drug consultation between physicians and pharmacists is conducted for patients taking anticoagulants, as well as a health education for patients on how to stop taking medicine that may lead to bleeding.

### 2.3. Proposed Scheme

#### 2.3.1. Scope of Data Use

##### Time

**Figure 1:**
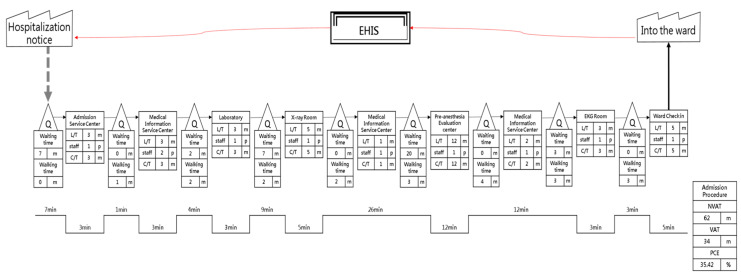
Current value flow chart for the hospital admission procedure.

The main objectives of the present study were to “improve hospital admission procedures for surgical patients, reduce unnecessary waiting times, optimize the quality of hospital services, and improve inpatient satisfaction”. By accompanying patients in the hospital and following the steps and time spent by patients during the hospital admission process, we were able to assess value-added time and non-value-added time, creating both a detailed flowchart (Table 3) and current value flowchart (Figure 1) of the hospital admission procedure. These can then be used to identify wasted time and inefficiency which is occurring in the hospital admission procedure [14,15].

After obtaining the required time values from the current value flowchart, we used the process cycle efficiency method proposed by George (2002) to determine whether each process conformed to the Lean concept, calculating the PCE and PCED for each process to analyze the efficiency as a basis for improving the process sequence [16]. PCE is the ratio of value-added time in each process cycle and is used to calculate the ratio of value-added time to cycle time. It can effectively measure the operational efficiency of each process. PCED is the impact of individual processes on the overall PCE value. Its significance for the product and process is the impact on PCE if the step is removed [17].

In Table 4, the PCE and PCED results from patient hospital admissions are shown for the guidelines of the second Medical Information Service Center examination (PCED = 6.6667), the first Medical Information Service Center examination (PCED = 3.2258), the pre-anesthesia checkup (PCED = 1.8322), and the hospital admission procedure (PCED = 1.7784), which are the top four items of the PCED process. In addition, the PCEDs were all positive values, indicating that prioritizing the improvements of these operations will have a greater impact on the overall process cycle efficiency, as well as showing that these items are lower value-added processes.

##### Patient Satisfaction

Satisfaction is a tool to measure each consumer’s perception regarding products and services, work experience, quality of life, and quality of outdoor recreation. It is also a widely used indicator for measuring behavior. The concept of satisfaction can be applied to the healthcare industry as well to better understand the subjective experiences and feelings of healthcare consumers towards the quality of healthcare services. The Joint Commission on Accreditation of Healthcare Organizations (JCAHO) and National Committee for Quality Assurance (NCQA) in the United States both promote better quality and safer healthcare for patients and have included patient satisfaction as one of the necessary indicators for evaluating healthcare quality. Better quality healthcare services will increase patient satisfaction in them. Through collecting data concerning the satisfaction of hospital inpatients, the present study identified areas for both improvement and refinement in the hospital admission process.

#### 2.3.2. Procedures for Collecting Experimental Data

**Figure 2:**
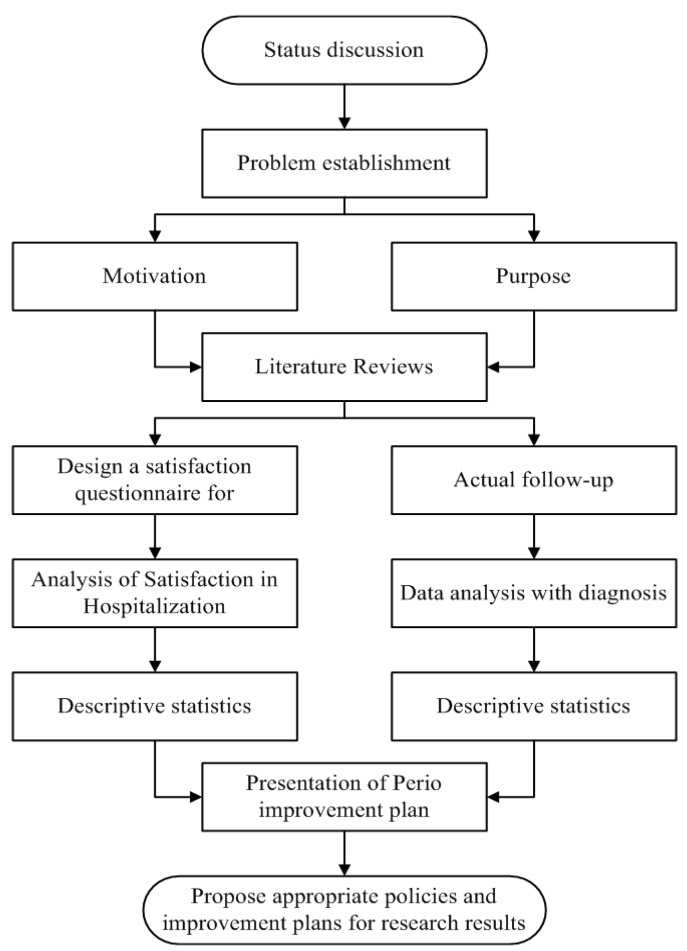
Research process.

The present study implemented the DMAIC framework to identify methods and activities useful for improving the hospital admission process at each stage and establishing the research process (Figure 2). The DMAIC framework was used in our study in the following manner:

(1). Define: The purpose of the present study was to shorten the time required for patients to complete hospital admission procedures, thus increasing the satisfaction of inpatients.
(2). Measure: The present study used a field survey that involved accompanying patients during the hospital admission process and recording the time spent on each of the hospital admission procedures.
(3). Analyze: The present study explored the reasons for repeating steps in the hospital admission process, as well as for increased waiting times.
(4). Improve: The present study proposed solutions based upon the results of the analysis, including the integration of equipment, refinement and optimization of processes prior to hospital admission, and information systems intervention (flow system, electronic tags, and app positioning and directions).
(5). Control: The present study used methods to control and improve the time required for hospital admission procedures in order to reach the desired level, including the establishment of Lean standard operating procedures, as well as the regular monitoring of relevant indicators for the continued improvement of patient satisfaction.

#### 2.3.3. Data Analysis Method

With regards to the patient satisfaction questionnaire data, we used SPSS 12.0 statistical software for coding, inputting, and removing errors, and subsequently analyzed the preliminary research results using descriptive statistics.

#### 2.3.4. Research Process

##### Process Re-Engineering in Conjunction with the Building Renovation Project

According to the actual walking route map and spatial positions of the units that patients came into contact with during the hospital admission procedure, the testing and examination units (such as the testing facilities, X-ray examination facilities, and ECG examination facilities), as well as the Pre-anesthesia Evaluation Center, were located on different floors within three buildings. This meant that patients dedicated a lot of time walking between units. Moreover, because the units were spread out, flow for the hospital admission process was poor, requiring patients to frequently return to the Medical Information Service Center in order to ensure that they would reach the next correct unit.

**Figure 3:**
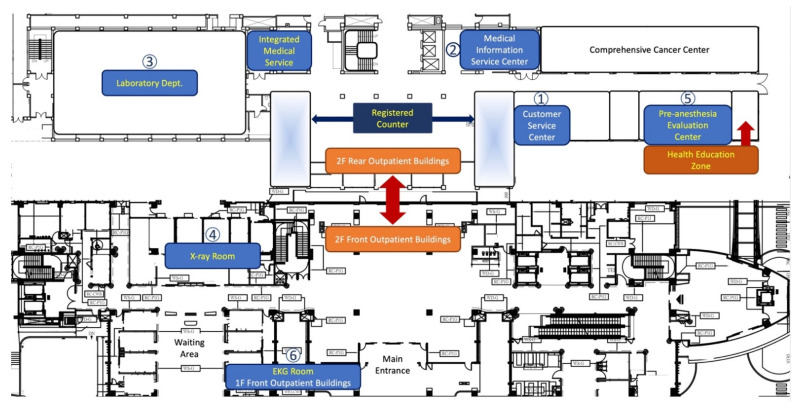
Floor Plan Following Renovation of the Rear Outpatient Building.

To improve the service provided by the Medical Service Center, the physical space required for examination and testing during the hospital admission process was integrated into the second floor of the Rear Outpatient Building, using this adjustment to optimize the inpatient process and deliver a one-stop hospital admission service. The floor plan for the hospital admission procedure following this renovation is shown in Figure 3.

The renovations were completed and put to use over the course of 2019. The spatial distribution of the units was changed from different floors of three different buildings to the first and second floors of the Front Outpatient Building and Rear Outpatient Building.

##### Re-Examination of the Hospital Admission Service Process

Surgery inpatients often need to undergo a pre-admission examination in the afternoon of the previous day, prior to surgery the following day. In these cases, the patient will need to be hospitalized for an additional day. After patients are admitted, the results of the pre-anesthesia checkup often do not either meet the criteria necessary for surgery or the assessment of the anesthesiologist, meaning that the results had to be reassessed by a different department. This results in the cancellation of the surgery and an end to the patient’s hospital stay, thereby affecting patient safety and subsequently leading to a waste of medical resources.

**Figure 4:**
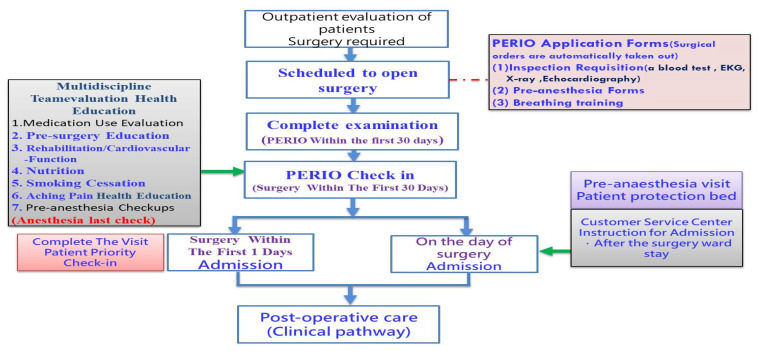
The present study established a perioperative assessment process.

The present study established a perioperative assessment process (hereafter referred to as the PERIO process, as shown in Figure 4) in order to improve the hospital admission process. The subjects involved were patients scheduled to undergo routine surgery in the orthopedics, urology, colorectal surgery, and otolaryngology departments. The patients completed all tests and examinations, pre-surgery education, drug consultations, and pre-anesthesia checkups the day prior to hospital admission and surgery.

In addition, assessment of the health education program by a cross-department team was added to the PERIO process, including such items as nutrition education, rehabilitation education, and respiratory training according to individual needs. This was done to improve safety during surgery, facilitate the recovery of patients, and reduce the average length of each hospital stay.

Information Systems Development

(1) Outpatient

“Enter the PERIO process” was added to the surgery scheduling function of the “Outpatient Physician System”. The outpatient physician determines when the patient needs to be admitted to hospital for surgery, and can therefore choose whether or not to have the patient undergo the PERIO process.

The HIS system can automatically open the schedule for testing and examination packages, as well as pre-anesthesia assessments for surgical departments. Outpatient physicians can also assess the need for additional tests based upon the patient’s condition.

(2) PERIO Check-In

After the outpatient physician selects the PERIO process for each patient, the “PERIO surgery query function” can then be used to query information, such as the patient’s scheduled PERIO date, surgery date, surgery time, and completion status.

The information system is used to manage the patient’s integrated perioperative assessment to ensure that all tests and examinations, pre-surgery education, drug consultations, and pre-anesthesia checkups are completed prior to surgery.

(3) PERIO Flow System

As part of the operating system, the PERIO Flow System function was added to the “Healthcare Information System” in order to assist the staff of the Customer Service Center with the check-in process when performing the patient’s integrated perioperative assessment. After the evaluation is completed, the Medical Information Service Center, Drug Consultation Room, group health education and Pre-anesthesia Evaluation staff all access the system to both confirm checkpoints and update the completion status.

The “PERIO Flow System” function can query the execution status of the testing and examination items that the patient must complete prior to surgery, which is helpful when the information system is out of sync. Nurses at the Medical Information Service Center can then immediately check which examination items have been completed by the patient and therefore do not require the querying of medical records separately.

(4) Electronic Tags, App Positioning and Directions Function

The present study introduced electronic tags to replace paperwork, showing the locations where each patient needs to complete the items listed in the integrated perioperative assessment seen on the tag. This combines the checkpoint-confirmation functions of the PERIO Flow System and updates the patient’s completion status for each item listed in the electronic tag. In addition, by downloading the TCVGH Mobile Registration app on their mobile phone and scanning the QR-Code on the electronic tag, each patient can use the hospital map positioning and guidance function to reach the location where each process takes place, thus reducing unnecessary walking time.

#### 2.3.5. Patient Satisfaction

The questionnaire used content validity and involved three experts with relevant backgrounds who reviewed the suitability of the questionnaire content. Appropriate adjustments based upon the opinions of the experts were performed in order to ensure that the questionnaire was optimal for the needs of the respondents. In addition, 30 patients were randomly selected for pretesting. After testing, the reliability α for the internal consistency of the questionnaire was 0.97.

The questionnaire was provided by the researcher in an electronic format on an iPad for respondents to complete at the Customer Service Center. Questionnaire respondents were patients visiting the Customer Service Center during hospital admission. We excluded minors (those younger than 20 years), patients with reduced mobility who required mobility aids and vulnerable groups (prison inmates, pregnant women, persons with disabilities, the mentally ill, and others who were determined by the review committee as being unable to make decisions of their own free will).

## 3. Results

### 3.1. Tangible Results

**Figure 5:**
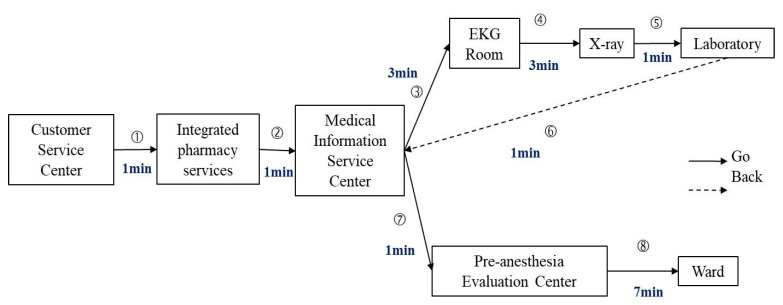
After the integration of the facility’s hospitalization procedure.

After integration of the facilities, each patient spent 73 min on average to complete the hospital admission procedures, from entering the Customer Service Center to arrival at the nurse’s station on the ward (Table 5). Compared to the average time of 96 min for the hospital admission procedures prior to the improvements, the integration of facilities saved 23 min in time required for the hospital admission procedure to be completed for orthopedics (Figure 5).

**Figure 6:**
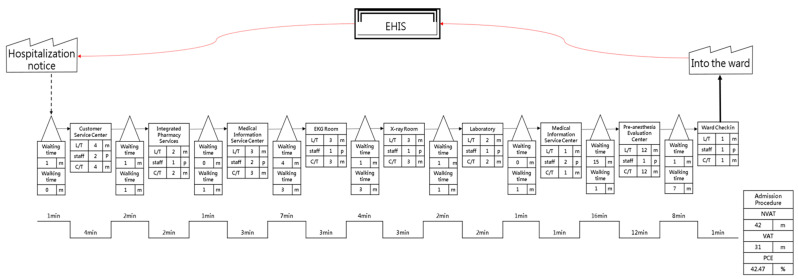
Future value flow chart for the hospital admission procedure.

We collected data on each of the steps needed for, and time spent on, the hospital admission procedure and assessed both the Value-added time (VA) and Non-value-added time (NVA), creating both a detailed flowchart (Table 6) and a future value flowchart (Figure 6) for the hospital admission procedure.

The present study incorporated the Lean Six Sigma as an improvement tool and applied it to the hospital admission procedure. Based on the evaluation of the impact that the improvement plan had on process times, the future value flowchart for the time spent on hospital admission procedures shows an improvement, as summarized in Table 7. PCE increased from 35.42% before improvement to 42.47%, an increase of 7.05%; VA was reduced from 34 min before improvement to 31 min, a slight decrease of 8.82% (3 min); non-value-added time was reduced from 62 min before improvement to 42 min, a significant reduction of 32.26% (20 min).

### 3.2. Patient Satisfaction

The present study designed a “Patient Admission Process Service Satisfaction” questionnaire for inpatients. The content included four dimensions: “basic information”, “satisfaction with hospital admission procedures during visit”, “satisfaction with the clear signs and directions seen on each floor of the hospital”, and “overall satisfaction with the hospital”. There were 21 question items in total. Aside from the six “Questionnaire data” items, the remaining 15 items were measured using a Likert scale, with scores ranging from 5 to 1 for “very satisfied,” “somewhat satisfied”, “neither satisfied nor dissatisfied”, “somewhat dissatisfied”, and “very dissatisfied”.

The pretest for the satisfaction questionnaire was implemented in April 2019, with the posttest-satisfaction questionnaire implemented between September and November 2019. A total of 450 questionnaires were collected. After the incomplete and invalid questionnaires had been excluded (7 for the pretest and 18 for the posttest), there remained 425 valid questionnaires (97 for the pretest and 328 for the posttest) with a recovery rate of 94.4%.

#### 3.2.1. Analysis of the Level of Satisfaction

The present study used SPSS 12.0 statistical software to conduct independent sample *t*-tests for the average satisfaction results taken from the pretests and posttests.

Average patient satisfaction in the pretest was 4.29, while the average patient satisfaction in the posttest was 4.40. The posttest satisfaction results for each item were higher than those seen in the pretest satisfaction results, with the difference ranging from 0.02 to 0.26. Of these, there was a significant difference in patient satisfaction (*p* < 0.05) for the following items: “the process of receiving an examination (X-ray, electrocardiogram) or test (blood, urine) is convenient,” “satisfaction with the health education guidance and consultation provided by healthcare personnel”, and “satisfaction with the pre-anesthesia assessment and consultation”. (Table 8)

The eight items on the “satisfaction with hospital admission procedures on this visit” did not show a significant difference between the pretest and posttest. Therefore, it remains necessary to re-examine whether any further improvements can be made to the relevant steps in the process.

#### 3.2.2. Additional Benefits

##### Average Length of Hospital Stay

We counted the number of hospital stay days for patients having surgical procedures who had experienced PERIO in the orthopedics, urology, colorectal surgery, and otolaryngology departments. This count was also performed for the same surgical procedures in 2018, prior to the implementation of PERIO, for purposes of assessing whether there was a difference in the average length of hospital stay before and after the introduction of the new processes. This was done in order to determine the effectiveness of the improvements made in the process.

**Figure 7:**
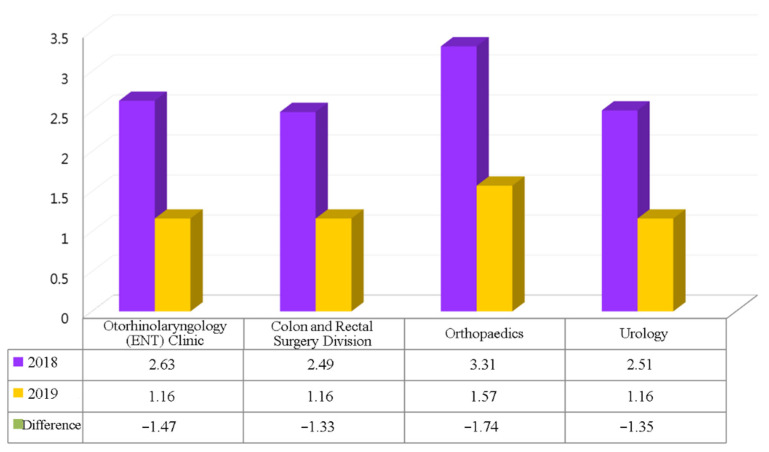
Average length of hospital stay.

As shown in Figure 7, the average length of hospital stays in 2019 was lower for both the departments and the surgical procedures that had introduced PERIO than it was for the same departments and surgical procedures in 2018. The difference was in the range of 1.33–1.74 days. Based on the number of cases (304 patients) using PERIO, the aggregate length of hospital stays was reduced by 472 days in 2019.

## 4. Discussion

Quality and efficiency are increasingly being used as indicators for satisfaction felt by surgical patients. Only hospitals which can consistently deliver high quality care and patient satisfaction at an affordable price can maintain financial viability. This study demonstrated the use of the smart Lean method for conducting high-quality integrated perioperative management in order to reduce patient waiting times, improve patient satisfaction and reduce the length of hospital stays.

Surgical patients often undergo testing, examinations, and pre-anesthesia checkups which are related to their surgery when they carry out hospital admission procedures on the day prior to surgery. As a result, excessive time is spent going through the hospital admission procedures. Additionally, a patient’s hospital admission and surgery are also canceled if the patient does not pass the necessary testing, examination surveys, or pre-anesthesia checkups.

The integrated preoperative assessment routine is one of the major entryways to hospitals for surgical patients. This clinic allows for the coordination of preoperative surgical anesthesia, nursing and laboratory care, preoperative medical optimization of patients, and transmission of information to the surgical team. However, the length of time required before being seen in the pre-anesthesia office, as well as printable cancellations and delays due to perioperative issues, have been shown to inversely correlate with patient satisfaction. To achieve the objective of shortening the time spent by patients during hospital admission procedures, the present study sought improvements in three areas: manpower, processing, and information systems.

(1). In terms of manpower, in conjunction with the building renovation project, the original Admission Service Center was merged with the Customer Service Center, ultimately increasing the manpower available at the service counter, while providing support during the hospital admission process at peak hours.
(2). In terms of processing, the integrated PERIO process ensured that patients completed health education, a smoking cessation course, all pre-operative tests, and the pre-anesthesia assessment in a shorter period of time.
(3). Building an information system, from the period of arranging the surgery during outpatient visits to implementing the patient’s integrated perioperative assessment, reduced the possibility of human error, while also providing the relevant functional units with instant patient information and data for statistical analysis for use as a reference for subsequent refinement.

Lean methods were first implemented by Womack and Jones in the 1980s as part of Toyota’s manufacturing process and have since been increasingly used in a health care setting to eliminate waste and improve value. This study, through both the re-design of space and integration of manpower, reduces a patient’s walking and waiting times. The results show that the percentage of Process Cycle Efficiency (PCE) increased from 35.42% to 42.47%, Value Added reduced from 34 min to 31 min, and Non-Value-Added reduced from 62 min to 42 min. In the hospital admission satisfaction survey, aside from the items, “the process of receiving an examination (X-ray, electrocardiogram) or test (blood, urine) is convenient”, “satisfaction with the health education guidance and consultation provided by healthcare personnel”, and “satisfaction with the pre-anesthesia assessment and consultation”, all the other items showed significant patient satisfaction. However, there still remains room for both additional review and improvement in order to enhance both the quality of healthcare and patient satisfaction [1].

Day of Surgery Cancelation (DoSC) represents a costly waste of Operating Room (OR) time and causes inconvenience, emotional distress, and a financial cost to families. Perioperative management (PERIO) procedures can bring greater value to hospitals by allowing them to focus their efforts on following evidence-based management on the day of surgery when cancellations or add-on cases disrupt the planned OR schedule, rather than on trying to establish clinical pathways for reducing inpatient cancellations [18,19]. Implementing the PERIO system may cause cancelations to reduce from 1.3% to 0.2%.

In this study, an integrated perioperative assessment was used to address the issue of pre-operative integration. Patients are required to properly complete all tests and examinations, as well as the pre-anesthesia assessment before the hospital admission procedures for surgery can be carried out. The PERIO process successfully reduced the time period necessary from admission to surgery and may reduce incidences of surgery cancellation. Perioperative management of blood sugar levels, heart function, and coagulation monitoring is important for minimizing any complications related to surgery [8,20,21,22]. The average length of a hospital stay for each patient after departments introduced PERIO was reduced by 1.33–1.74 days. These results were similar with Bianchini C et al., in that there was reduced morbidity, improvement in recovery, and shortened hospital stays for surgical patients [23,24,25,26].

## 5. Conclusions

The present study implemented the DMAIC architecture of the Lean Six Sigma as a tool for improving its hospital admission procedures. Integrating different units and establishing standard operating procedures, together with the support of information systems, all helped shorten the time spent by patients during hospital admission. At the same time, these changes improved the comprehensiveness of the preoperative preparations, as well as the surgical safety of patients, thereby facilitating the provision of high-quality healthcare services. In addition, reducing the average length of hospital stays can help clear beds and increase the turnover of patients, thus benefiting the overall operations of the hospital. Therefore, adopting a patient-centered approach in order to achieve a leaner process that strengthens improvement at the policy, system, and management levels can effectively improve the quality of hospital services and patient satisfaction, ultimately achieving a win-win result for both doctors and patients.

## Figures and Tables

**Table 1:** Inpatient Satisfaction Survey Report during the years 2015 to 2017.

Questionnaire Items	2015 (1) (*n* = 573)	2015 (2) (*n* = 525)	2016 (1) (*n* = 490)	2016 (2) (*n* = 455)	2017 (1) (*n* = 437)	2017 (2) (*n* = 424)
6. Processes for various examinations during hospital admission	4.55	4.53	4.59	4.59	4.61	4.56
Average level of satisfaction	4.58	4.57	4.61	4.62	4.65	4.58
18. Convenience of hospital admission procedures at the Admission Service Center	4.20	4.18	4.29	4.31	4.39	4.31
Average level of satisfaction	3.59	3.54	4.18	4.19	4.26	4.21
23. Overall, based upon your experience at this hospital, how would you rate our services?	4.59	4.58	4.58	4.63	4.63	4.59
The average to all customer satisfaction	4.44	4.44	4.48	4.55	4.52	4.49

**Table 2:** Five stages of the DMAIC.

Define	The first phase involves defining the problem and mapping it out.
Measure	The second phase of DMAIC involves determining the project’s main metrics. How will they be measured?
Analyze	In the third phase of DMAIC, you look at data from your current process to see how stable or capable it is. Your operation or process must be able to produce results (products) that meet the specified requirements, so this is where you evaluate the standard deviation as compared to the specification limits (tolerance limits).
Improve	In the previous phase, you identified which of the factors have an impact on Y. In this phase you’ll investigate how changes in X will impact the result.
Control	The focus of this phase is to ensure that the performance objective you identified in the Improve phase is well implemented and maintained.

**Table 3:** Detailed process of the hospitalization procedures.

Process	Item	VA/NVA	Time (min.)
Instructions for Admission	Waiting for your number to go through hospitalization	NVA	7
	Completion of hospitalization consent	VA	3
Medical Consultation	Move to Medical Information Service Center	NVA	1
	Measuring height and weight	NVA	1
	Confirm the inspection items and explain the location of the inspection unit	VA	2
Laboratory Dept.	Move to inspection station	NVA	2
	Waiting	NVA	2
	Blood test	VA	3
X-ray	Move to X-ray examination room	NVA	2
	Waiting to be called	NVA	7
	X-ray	VA	5
Medical Information Service Center Check boot (1)	Move to Medical Information Service Center	NVA	2
	Directed to the next check unit	NVA	1
Pre-anesthesia Checkups	Move to Pre-anesthesia Evaluation Center	NVA	3
	Complete the self-assessment form	VA	4
	Height, weight, and blood pressure blood oxygen measurements	VA	2
	Waiting to be called	NVA	20
	In the Clinic Room Pre-Anesthesia Assessment	VA	6
Medical Information Service Center Check boot (2)	Move to Medical Information Service Center	NVA	4
	Guide to the next inspection unit	NVA	2
EKG Room	Move to EKG Room	NVA	3
	Wait for your number to be called	NVA	3
	EKG Room	VA	3
Ward Check in	Move to nurse’s station	NVA	3
	Check-in	VA	4
	Move to bed	NVA	1
Hospitalization cycle time (Total)			96

**Table 4:** Process-cycle loss-efficiency analysis table.

Process	Walk Time	Wait Time	Operating Time	VA	NVA	Cycle Time	PCE	PCED
Hospitalization	0	7	3	3	7	10	0.3605	1.7784
Medical Information Service Center	1	0	3	3	1	4	0.3370	−4.8593
Laboratory Blood	2	2	3	3	4	7	0.3483	−1.6523
X-ray Room	2	7	5	5	9	14	0.3537	−0.1435
Medical Information Service Center Check (1)	2	0	1	0	3	3	0.3656	3.2258
Pre-anesthesia checkups	3	20	12	12	23	35	0.3607	1.8322
Medical Information Service Center Check (2)	4	0	2	0	6	6	0.3778	6.6667
EKG Room	3	3	3	3	6	9	0.3563	0.6085
Ward Check-in	3	0	5	5	3	8	0.3295	−6.9519
Hospitalization time (Total)				34	62	96	0.3542	

**Table 5:** The average time for patients to complete the hospitalization procedure after hardware integration.

Item	Pre-Test (*n* = 40)	Post-Test (*n* = 44)
Total work time	74	55
Customer Service Center Hospitalization	10	5
Integrated Pharmacy Services	-	3
Medical Information Service Center	5	3
EKG Room examination	6	7
X-ray	12	4
Laboratory	5	3
Pre-anesthesia Checkups	32	27
Ward Check in	4	2
Total walk time	42	18
Total hospitalization cycle time	96	73

**Table 6:** Detailed flow chart for hospitalization procedures.

Process	Item	VA/NVA	Time (min.)
Instructions for Admission	Waiting for your number to go through the hospitalization process	NVA	1
	Completion of hospital consent and measurement of height and weight	VA	4
Drug consultation	Move to Comprehensive Drug Consultation	NVA	1
	Drug consultation	VA	3
Medical consultation	Move to Medical Information Service Center	NVA	1
	Confirm the inspection items and explain the location of the inspection unit	VA	3
EKG	Move to EKG rooms	NVA	3
	Wait for your number to be called	NVA	4
	EKG examination	VA	3
X-ray	Move to X-ray rooms	NVA	3
	Wait for your number to be called	NVA	1
	X-ray	VA	3
Laboratory	Move to Laboratory	NVA	1
	Queue wait	NVA	1
	Draw blood	VA	2
Medical consultation guidance	Move to the Medical Information Service Center	NVA	1
	Guide to the next inspection unit	NVA	1
Pre-anesthesia Checkup	Move to Pre-anesthesia Evaluation Center	NVA	1
	Wait for your number to be called	NVA	15
	Pre-anesthesia Visit Required	VA	12
Nurse Station Check In	Move to ward	NVA	7
	Check in	VA	1
	Move to bed	NVA	1

**Table 7:** Benefit evaluation before and after improving six standard deviations.

Benefit	before Improvement	Improved	Benefit
VA	34	31	3 min shorter
NVA	62	42	20 min shorter
PCE	35.42%	42.47%	Increase of 7.05%

**Table 8:** Analysis of the Level of Satisfaction.

Questionnaire Items	Pre-Test (*n* = 97)	Post-Test (*n* = 328)	*p* Value
The average to all customer satisfaction	4.29	4.40	*p* < 0.05
Processes for various examinations during hospital admission	4.18	4.44	*p* < 0.05
Pre-operative anesthesia evaluation and counseling	4.30	4.47	*p* < 0.05
Medical staff provide health education guidance and consultation	4.26	4.42	*p* < 0.05

## Data Availability

The datasets used and/or analyzed during the current study are not publicly available due to General Data Protection Regulations; however, they are available from the corresponding author upon reasonable request.

## Abbreviations

VA: Value-Added; NVA: Non-Value-Added; PCE: Process Cycle Efficiency; PCED: Process Cycle Efficiency Destruction; LOS: Length of Stay; DMAIC: Define, Measure, Analyze, Improve, Control; TCVGH: Taichung Veterans General Hospital; PERIO: Perioperative Management; JCAHO: Joint Commission on Accreditation of Healthcare Organizations; NCQA: National Committee for Quality Assurance.
